# Dietary Sources of Sodium in Nigerian Adults From 3 Geographic Regions: A Population-Based Cross-Sectional Study

**DOI:** 10.21203/rs.3.rs-5829587/v1

**Published:** 2025-01-15

**Authors:** Aniekeme George, Dike Ojji, Anthony Orji, Felix Adurosakin

**Affiliations:** University of Oxford; University of Abuja; University of Abuja Teaching Hospital; Federal Ministry of Health

**Keywords:** Dietary sodium, sources of sodium, dietary recall, Nigeria

## Abstract

**Background:**

To address the growing burden of hypertension and related diseases, Nigeria seeks to reduce excess dietary sodium through policymaking. The current study aims to describe the levels and sources of dietary sodium intake among Nigerian adults to inform targeted policies for reducing sodium intake.

**Methods:**

From June 2023 to July 2023, adults aged 18 to 70 years old were recruited from the Federal Capital Territory, Kano States, and Ogun States to participate in a population-based, cross-sectional non-communicable diseases survey. Data were also collected to assess levels and dietary sources of sodium through four 24-hour dietary recalls by trained study personnel with 90.7% response rate. Concurrent 24-hour urine sodium data were collected. The primary analyses included the distribution of sodium intake and sources of sodium, overall and by sex and state. Results were adjusted to the Nigerian population. Multivariate regression models evaluated associations between baseline sociodemographic factors and sodium intake.

**Results:**

Among 537 participants, 365 (68.0%) were female, and median (Interquartile range) age was 38 (27, 48) years. Adjusted median (IQR) daily sodium intake according to 24-hour dietary recalls was 3,803 (2,663, 5,085) mg per day with higher intake reported among males (males: 3,878 [2,663, 5,032] mg/dl; females: 3,415 [2,373, 4,689], p<.0001). Two-thirds (67.0%) of the sodium intake was from home-cooked meals. Nearly half (48.7%) of sodium came from discretionary sources, including 21.4% from bouillon. Salt and yaji spice added at the table accounted for 9.1% of sodium intake and was highest among females (18.8%) and males (13.7%) in Kano. On the other hand, sodium from street food was highest in males (35.9%) and females (34.2%) in Ogun. After adjustment, older participants and those with higher education had lower daily sodium intake compared to younger participants and those with less education, respectively. Results were similar when excluding individuals with cardiovascular disease or hypertension.

**Conclusions:**

Adults in the Federal Capital Territory, Kano, and Ogun consume nearly twice the recommended level of dietary sodium. Most dietary sodium intake came from home cooked foods, nearly half of which came from discretionary sources, which has important policy implications for SHAKE package implementation.

## INTRODUCTION

High dietary sodium intake is a significant public health concern worldwide, contributing to the global burden of non-communicable diseases (NCD) such as hypertension, cardiovascular diseases, and stroke.^1^ Healthy diets, particularly diets low in sodium, play an important role in cardiovascular health, with excessive dietary sodium consumption being a major risk factor for hypertension and hypertension related complications.^2,3^ In Nigeria, as in many other countries, excessive dietary sodium consumption is prevalent based on national estimates and modeling studies.^4–6^ Reducing dietary sodium intake by 30% from 2019 to 2025 is a major target outlined in Nigeria’s 2019 National Multisectoral Action Plan for the Prevention and Control of Noncommunicable Diseases^5^ through implementation of the World Health Organization’s SHAKE technical package.^7^

While numerous studies have investigated sodium intake in population-based samples^1,8,9^, including Nigeria^6^, fewer have evaluated the dietary sources of sodium^10^, and none, to our knowledge, among Nigerian adults. Understanding the sources of dietary sodium is crucial for developing effective interventions to reduce sodium intake and mitigate associated health risks.^11^ Given the diverse cultural and dietary practices within Nigeria, regional variations in sodium sources are also expected. Therefore, identifying these sources is essential for tailoring multi-level strategies to promote healthier dietary habits and reduce excessive dietary sodium intake at the population level. It is also recommended by the World Health Organization to guide evidence-informed policymaking.^1^

The Nigeria Sodium Study includes three waves of retail surveys^12^, stakeholder interviews^13^, and population surveys to evaluate the implementation and effectiveness of national dietary sodium policies in Nigeria. The current study aims to examine the sources of sodium in the diets among the first wave of a population-based sample of Nigerian adults from three distinct geographic regions: Federal Capital Territory (North Central), Kano State (Northwest), and Ogun State (Southwest). The study investigated the levels, sources, and differences in dietary sodium intake among Nigerian adults from these regions across baseline sociodemographic and clinical characteristics. By elucidating the sources of sodium in the diets of Nigerian adults across diverse geographic regions prior to national sodium policy implementation, this study will provide valuable baseline, and ultimately follow-up, information for policymakers, public health professionals, and dieticians. These insights will aid in reducing sodium intake, promoting healthier dietary patterns in Nigeria, and ultimately decreasing the burden of diseases related to excessive dietary sodium intake through targeted interventions.

## METHODS

### Population and Recruitment

Recruitment was conducted among participants of the STEPwise approach to NCD risk factor surveillance (STEPS) cross-sectional survey, which was led by the Nigerian Federal Ministry of Health and supported by the World Health Organization Nigeria office. STEPS participants were recruited using a multi-stage representative sampling frame and simple random sampling without replacement. In brief, 12 households from 60 clusters were selected to complete the STEPS survey and provide biological samples in each of the 36 states in Nigeria plus the Federal Capital of Territory. Additional participants were recruited to account for an anticipated 20% cumulative non-response rate.

As a sub of the main STEPS survey, from June 2023 to July 2023, a target sample of 450 adults aged 18 to 70 years old was recruited from 3 geographic locations (states): Federal Capital Territory (target n = 150), Kano (target n = 150), and Ogun (target n = 150) among STEPS participants for the sodium study. The study design, sample size, and methods for the dietary sources of sodium study was modeled after a previous study conducted in the United States.^11^ Additional participants were recruited to account for anticipated dropout across multiple dietary recall interviews. Participants who were pregnant or breastfeeding were excluded based on differences in dietary intake. All participants provided written informed consent. The institutional review boards at University of Abuja, Northwestern University, and University of New South Wales approved the study procedures.

### Overview of Data Collection

Data collection activities for the STEPS survey were performed by trained research staff from the Federal Ministry of Health or World Health Organization Nigeria office and supported by Resolve to Save Lives. Data collection activities for the dietary sources of sodium study were performed by trained dietitians recruited by the Cardiovascular Research Unit of University of Abuja and University of Abuja Teaching Hospital following extensive training and certification overseen by the University of Minnesota Nutrition Coordinating Center. Data collection occurred in person, often in participants’ homes. Bar codes, unique to each participant, were used to ensure accuracy of harmonization of participant data across multiple data sources.

### STEPS Data

Study dieticians administered questionnaires to collect data on sociodemographic, health behaviors, medical history, and medication use. Height was measured in centimeters using a stadiometer, and weight was measured in kilograms using a digital scale. Participant report of prior heart attack, angina, or stroke were used to indicate a self-reported history of cardiovascular disease. Point of care devices were used to evaluate blood glucose and cholesterol.

### Dietary Sources of Sodium Data

In addition to the questionnaire and biological samples provided in the STEPS survey, participants in the current study were invited to complete 4 separate 24-hour dietary recalls over the course of 1–2 weeks, including 1 recall on a weekend day, and to provide a 24-hour urine sample. Participants were compensated ₦500 (~ 33 US Cents) for survey completion, ₦6000 (~ 4 US Dollars) for participation in 4 dietary recalls, and ₦3000 (~ 2 US Dollars) for 24-hour urine collection.

Dietitians interviewed participants using a multiple passe approach to obtain their 24-hour diet history. Participants were shown a standardized food atlas with pictorial representation of portioned meals, and household cooking measures and an electronic weighing scale were used to estimate their amount of food intake during each recall. Responses provided by participants were documented by the dietitians in a 24-hour dietary recall form. Recipes for foods prepared at home that had salt or salty seasonings as an ingredient were also collected from the household member who prepared the meal, including serving sizes, number of servings, and total yield (i.e., food prepared for the family). For every food item eaten, participants were asked to report the food source as home, restaurant, or street food, or other settings. Other settings were defined as meetings, events/occasions, parties, friends’ and relations’ homes, etc. For each meal, participants were asked if they added any salt or salty seasoning to their food at the table. If they had added salt or a salty seasoning at the table, they were asked to estimate the amount added.

### Duplication Samples of Salt Data

In conjunction with each diet recall, bagged salt samples ranging from 1 g to 24 g were provided for participants to estimate the quantity of salt that was added in home cooked meals and at the table for each 24-hour period. Estimates were validated by requesting the household member who prepared the meal to estimate the quantity of salt added while cooking using a 1 kilogram sachet of salt that the dietitians provided as a reference. In addition, household members were asked to estimate the quantity of bouillon and seasonings used in preparing foods, and the net weights of these were recorded and validated by weighing them on an electronic scale.

### 24-Hour Urine Data

Participants were provided training to collect a 24-hour urine sample by trained medical laboratory scientists and directed to collect the sample approximately 3 days after initial enrollment. Samples were collected from participants to overlap with one of the 24-hour dietary recalls as much as feasible for comparison with estimated sodium intake. Urine samples were processed using a standardized approach and analyzed as International Organization for Standardization-certified laboratories in Abuja and Lagos, Nigeria.

### Procedures for Calculating Sodium Intake by Source

The Nutrition Data System for Research (NDSR) software (version 2022), developed by the Nutrition Coordinating Center at the University of Minnesota of Minneapolis, MN, was used for calculating energy and sodium intake for the 24-hour dietary recalls.^14^ Prior to entering the dietary recalls into NDSR for nutrient calculation, recipes common to Nigeria were added to the software by local nutrition experts. Using data available in NDSR output files, sodium intake from the following were quantified for each 24-hour dietary recalls: 1) salt or salty seasonings (e.g. bouillon) added to food during home preparation, 2) salt or salty seasonings added to food at the table for each location (home cooked, restaurant, street food, and other), and 3) sodium inherent in food or added in commercial processing for each location. The rules and procedures used to determine the proportion of sodium from each of these sources based on previous research.^11^ To calculate sodium intake accurately, the dietary recall entry procedure included the following steps: (1) entering food source codes into the NDSR to identify the sources of sodium; (2) matching packaged foods and foods from the Nigerian Food Database 2019 to foods in the NDSR that were within 85 kcal and 50 mg of sodium per 100 g; (3) creating user recipes for foods that could not be matched to an existing food in the NDSR; (4) entering water data according to rules that reflected sodium levels depending on the water source; (5) coding extra salt added while eating with a user recipe named ‘Extra salt added while eating (NaSS001)’ and (6) entering household recipes when provided by participants in order to reflect the use of salt and other salty seasonings (e.g. bouillon) in home cooking. Once dietary recalls were entered into the NDSR and reviewed with a multi-step quality assurance process, NDSR output data files were generated, which provided the sodium intake calculations with the source codes for analysis.

### Statistical Analysis

Primary analyses are reported overall and by sex among participants with documented sociodemographic characteristics and at least one diet recall. Continuous variables are reported as median with interquartile range (IQR) because most data were skewed and differences by sex were assessed using Mann-Whitney U tests or Kruskal-Wallis tests, as appropriate. Categorical variables are reported as proportions and differences by sex were assessed using Chi-Square tests. We explored variability in sodium (mg/day) or energy (calorie/day) intake between weekdays and weekend days, but did not observe significant differences; therefore, we did not weight the data accordingly.

We report the number of recalls and calculated the overall and median sodium intake by location in which foods were consumed. We further calculated the number and proportion of dietary recalls that exceeded World Health Organization recommended daily sodium intake (2,000 mg), energy intake (calories), and sodium density as sodium (mg) per calorie (kcal). We will separately report sodium intake from packaged foods compared with sodium inherent in foods.

Urine samples from the Federal Capital Territory were standardized to 24 hours but start and stop times were not collected for samples in Kano or Ogun so no adjustments were made for these. All samples were converted from mmol per day to milligrams per day based on urine volume. A 0.95 conversation factor was applied to account for incomplete excretion of sodium in urine based on the International Consortium for Quality Research on Dietary Sodium/Salt.^15^ Some samples were excluded based on implausibly low urine volume (< 300 ml) or creatinine excretion levels (< 6 mmol/day for males and < 4 mmol/day for females) for the primary analysis using previously published thresholds.^16^ We conducted additional sensitivity analyses using alternative thresholds for 24-hour urine collection completeness to evaluate the robustness of these results. We created Bland-Altman and difference-in-difference plots to evaluate agreement between 24-hour urine sodium excretion and average diet recall sodium intake based on completeness as recommended by the International Consortium for Quality Research on Dietary Sodium/Salt.^17^

We evaluated sodium intake across subgroup categories by sex, including state, rurality (rural or urban), age (17–29 years, 30–44 years, 45–59 years, 60–70 years), level of educational attainment (none, less than primary school, primary school [grade 6], junior secondary school [grade 9], senior secondary school [grade 12], college/university, post-graduate), marital status (never, current or cohabitating, formerly), religion (Christian, Muslim, other), smoking status (current versus not current), alcohol use in the last 30 days, body mass index (underweight or normal, overweight, obese), blood pressure (< 140/90 mm Hg or ≥ 140/90 mm Hg) history of blood pressure measurement, hypertension, cardiovascular disease, and diabetes in bivariate tests and unadjusted regression analyses. Bivariate analyses were repeated and stratified by state and rurality to explore regional variation. The 2022 World Bank male and female population estimates^18^ and the 2018 Nigeria Demographic and Health Survey^19^ were used to weight calorie intake, sodium intake, and sodium excretion by sex, age, and rurality to estimate the population intake and excretion for adults aged 18–69 years.

Given the absence of significant clustering at both the state and cluster (i.e., geographic sampling unit) levels, indicated by an intraclass correlation coefficient of less than 5%, we conducted multivariable generalized linear regression analyses to evaluate potential differences in sodium intake across groups. Generalized estimating equations were utilized to account for multiple diet recalls per participant. Interaction terms between state and sex were included in adjusted models if P < 0.10. This threshold was chosen to control for type II errors in detecting interactions that could be important for policy generation. The overall estimated mean sodium intake (mg/day) was reported for each state-sex combination in models including an interaction term and all other estimates represent the additive effects to these state-sex estimates associated with specific characteristics. Models were adjusted for sex, state, age, education, and, in an additional sensitivity model, energy intake (calories). We also conducted an adjusted analysis to participants without cardiovascular disease or hypertension to minimize the risk of reverse causality in subgroup analyses.^20^

We conducted a complete case analysis and used a two-sided P < 0.05 to define statistical significance without adjustment for multiple testing. Analyses were conducted using SAS version 9.4 (SAS Institute, Cary, NC).

## RESULTS

**Figure 1:**
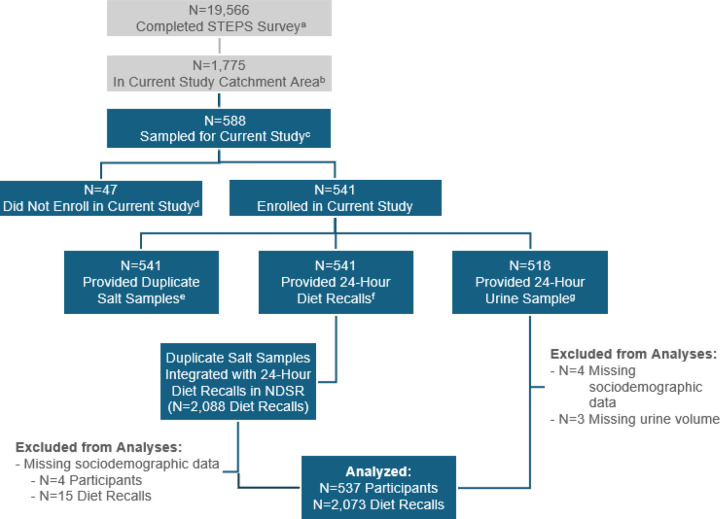
Flowchart of Participant Selection and Analysis. ^a^ Conducted by the Nigerian Federal Ministry of Health and World Health Organization Nigeria office ^b^ Includes Federal Capital Territory, Kano, and Ogun States ^c^ Random sample of n=49 Clusters from current study catchment area; n=12 participants per cluster ^d^ Declined (54.2%) or were unavailable (45.8%) to participate ^e^ Duplicate salt samples collected for salt added during home preparation and added at the table ^f^ Up to n=4 diet recalls per participant; at least n=1 recall on a weekend day ^g^ n=1 24-hour urine sample per participant Abbreviations: NDSR, Nutrition Data System for Research database; STEPS, STEPwise approach to NCD risk factor surveillance

Figure 1 shows the flow of participant recruitment and analysis. Among 19,56624 participants who completed the STEPS survey throughout Nigeria, 1,775 (9.1%) were from the Federal Capital Territory, Kano, or Ogun. Of these,588 (33.1%) were approached for the current study and 541 agreed to participate (92.0% of those approached) Four participants (0.1%) were excluded from analyses due to lack of sociodemographic data.

Table 1 shows the baseline sociodemographic and clinical characteristics overall and stratified by sex. Among the included study participants (n=537), 365 (68.0%) were female with a similar proportion of females recruited across the three states. Overall median (IQR) age was 38 (27, 48) years. Females had lower levels of education attainment (p=0.01) and annual household income (p=0.01), but a higher proportion were married or cohabitating (67.5% versus 60.2%) or formerly married (14.9% versus 4.1%, p<.0001). A larger proportion of males were current smokers (10.5% versus 1.1%, p<.0001) and alcohol users (26.2% versus 10.1%, p<.0001). Median (IQR) body mass index was higher among females compared with males (24.8 [21.2, 29.6] kg/m^2^ versus 22.0 [19.9, 24.5] kg/m^2^, p=0.002), but median (IQR) systolic blood pressure was lower among females (122.3 [112.3, 137.3] mm Hg versus 128.7 [118.7, 139.7] mm Hg, p=0.002). While 27.2% of participants who had a blood pressure measurement in the past self-reported having a history of high blood pressure, approximately one-third (32.4%) of all participants had high blood pressure (≥ 140/90 mm Hg) when measured at enrollment. Self-reported history of cardiovascular disease (15.1%) was also similar between sexes. Baseline characteristics by state and rurality are reported in Supplemental Tables 1a and 1b. Briefly, participants from the Federal Capital Territory (40.8%) were more likely to be urban, have a higher annual household income, higher body mass index, and higher diastolic blood pressure than the other locations. Participants from rural areas (14.5%) had less education, higher annual household income, higher tobacco and alcohol use, lower body mass index, and higher systolic and diastolic blood pressures.

Table 2 shows the daily sodium intake from the dietary recalls and 24-hour urine sample overall and stratified by sex. Most (90.7%) participants completed all 4 dietary recalls. Males reported a higher calorie (2,417 vs 2,058 kcal/day, p<.0001) and sodium (3,878 vs 3,415 mg/day, p<.0001) intake compared to females. When adjusted for the Nigerian population, the median (IQR) sodium intake was 3,803 (2,663, 5,085) mg/day. Similarly, a larger proportion of diet recalls reported by males exceeded the World Health Organization recommendation of <2,000mg sodium intake per day (87.5% vs 82.8%, p=0.01). Females reported a larger proportion of caloric (67.7%) and sodium (70.9%) intake inside the home compared to males (52.7%, p<.0001 and 56.5%, p<.0001, respectively) with the remainder of intake primarily from street foods among both sexes. Median (IQR) sodium excretion in 24-hour urine was 3,008 (2,070, 4,273) mg/day and was similar between sexes. [Assessment of 24-hour urine collection completeness is reported in Supplemental Table 3.] Results stratified by state and rurality are reported in Supplemental Table 2a and 2b, respectively. In brief, sodium intake was highest in Kano, followed by Ogun and Federal Capital Territory (4,005 vs 3,510 vs 3,209 mg/day, p<.0001, respectively). A higher proportion of sodium intake came from the home in Federal Capital Territory (73.8%) compared to Kano (67.8%) and Ogun (54.9%, p<.0001). Furthermore, participants in rural communities reported higher sodium intake (3,913 vs 3,470 mg/day, p<.0001), with 91.5% of diet recalls exceeding the World Health Organization recommended sodium intake (vs 83.1%, p=0.0002).

**Figure 2:**
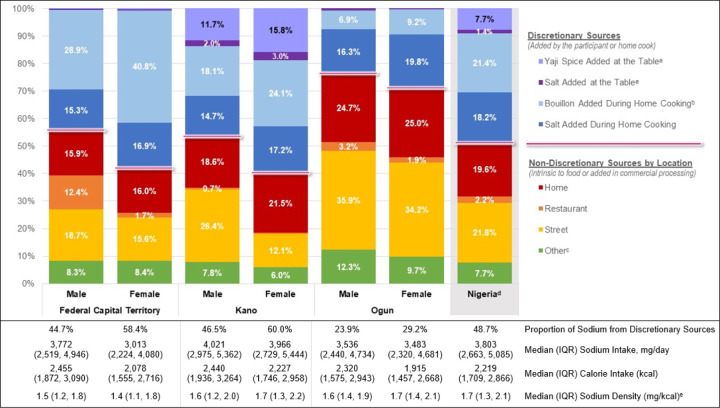
Proportion of Sodium Intake, mg/day, by Location of Food Source. ^a^ Added at the Home, Restaurant, Street, or Other Table ^b^ Includes Yaji Spice (Kano: Males: 1.6% Yaji vs. 16.6% Bouillon; Females: 2.1% Yagi vs. 22.0% Bouillon. 0% Yaji in Federal Capital Territory and Ogun.) ^c^ Captured as meetings, events/occasions, parties, friends’ and relations’ homes, etc. ^d^ Current study results weighted to reflect the Nigerian adult population (age 18–69) distribution by sex, age, and rurality ^e^ Sodium (mg) per calorie (kcal)

Figure 2 shows the proportion of sodium intake by location of food source, stratified by sex and state. Most sodium was derived from home cooking (including sodium inherent in food and added during commercial processing, salt added during preparation, and bouillon added during preparation) in both sexes in the Federal Capital Territory and Kano and among females in Ogun. Notably, sodium from bouillon added during home food preparation contributed to 40.8% of total sodium intake among females in the Federal Capital Territory. Salt and yaji spice, which includes salt and bouillon, added at the table was highest among females (18.8%) and males (13.7%) in Kano State. On the other hand, sodium from street food was highest in males (35.9%) and females (34.2%) in Ogun State.

**Figure 3:**
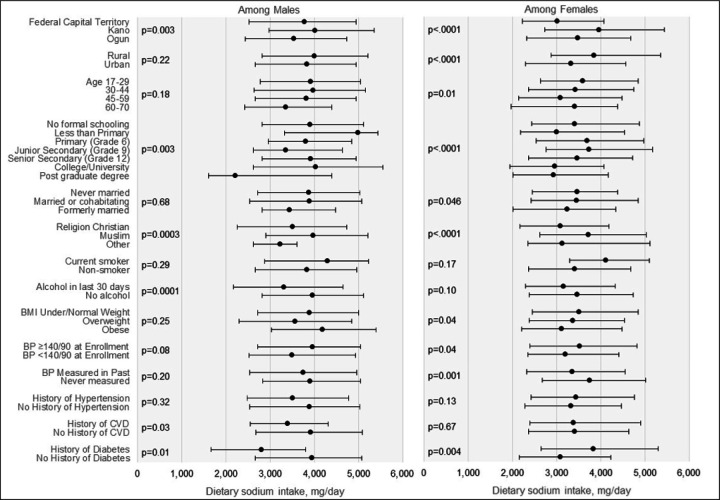
Median [IQR] Dietary Sodium Intake (mg/day) Reported by Diet Recall, by Sex. NOTE: P-values for bivariate tests (Mann-Whitney, Kruskal-Wallis, Chi-Square) comparing sodium intake across categories. Abbreviations: BMI, body mass index, kg/m^2^; BP, blood pressure; CVD, cardiovascular disease

Figure 3 shows median daily sodium intake by subgroups stratified by sex. Among males, there were differences in unadjusted sodium intake by state, level of education, religion, alcohol use, diabetes status, and cardiovascular disease status. Among females, there were differences in unadjusted sodium intake by state, age group, level of education, marital status, religion, body mass index, history of blood pressure measurement, hypertension, and diabetes.

The results of the unadjusted and adjusted regression models investigating sodium intake from diet recalls across sociodemographic characteristics are shown in Table 3. After adjustment for age and education, the difference in daily sodium intake between females and males varied by state (state-sex interaction terms p_Kano_=0.048 and p_Ogun_=0.03 versus Federal Capital Territory). The largest gender difference was observed in the Federal Capital Territory (−642.1 mg [95% CI: −924.2, −360.0]), with more modest differences in Kano (−191.4 mg [95% CI: −534.4, 151.5]) and Ogun (−140.1 mg [95% CI: −486.9, 206.7]). The highest daily sodium intake was among males in Kano (4,291 mg [95% CI: 3,962 mg, 4,620 mg]), and the lowest daily sodium intake was among females in the Federal Capital Territory (3,273 mg [95% CI: 3,065 mg, 3,482 mg]). Participants aged 60–70 years old had −351.2 mg (95% CI: −672.9 mg, −29.6 mg) lower daily sodium intake compared with participants 30–44 years old (p=0.03). Participants with post-graduate degree also had a −726.1 mg (95% CI: −1,239.3 mg, −212.9 mg) lower daily sodium intake compared with those who completed senior secondary school [grade 12]. Additionally, in unadjusted analyses, each additional calorie of intake was associated with 1.11 mg/day higher sodium intake (β = 1.11 [95% CI: 1.04, 1.18]). Results of sensitivity analyses adjusted for calories and sodium excretion in 24-hour urine and subgroup analyses among those without self-reported history of cardiovascular disease and without cardiovascular disease or hypertension are reported in Supplemental Table 4. In brief, sensitivity and subgroup analyses showed a similar direction and magnitude of associations after multivariate adjustment.

## DISCUSSION

This study used rigorous multi-pass dietary recall methods, along with concurrent 24-hour urine collection, to estimate sodium intake and sources of sodium among Nigerian adults in the Federal Capital Territory, Kano State, and Ogun State. Results demonstrate that adjusted median dietary sodium intake (3,803 mg per day) was nearly two-fold higher than the World Health Organization daily recommended level of 2,000 mg per day.^1,7^ More than 80% of dietary recalls in the current study had sodium intake greater than this recommended level. Dietary sodium intake was higher among males compared with females and was higher overall in Kano compared with the other two states. Older adults aged 60–70 years old and participants with post-graduate degrees had lower sodium intake compared with other groups. In contrast with results from high-income countries like the United States^11^ and consistent with Nigerian cultural practices, home cooked meals were the leading source of dietary sodium and calorie intake. These results also demonstrated a high proportion of dietary sodium from street foods, which are widely available and affordable, yet are also an underrecognized and largely unregulated source.^21^

Geographic variability in the dietary sources of sodium has been reported. For example, in a 2020 systematic review of 80 dietary sources of sodium studies conducted in 34 countries there was an inverse correlation between per capita gross domestic product and discretionary salt use.^10^ In this review, more than half of sodium intake comes from discretionary salt in low- and middle-income countries. However, none of these studies were from Nigeria, the most populous country in sub-Saharan Africa, with the only other sources of sodium study from Africa conducted in Mozambique among 100 hospital workers. In the Mozambique study, discretionary salt intake represented 60% of the daily sodium intake, which was estimated to be 4,220 (SD: 1,830) mg per day based on 24-hour urinary excretion.^22^

Previous epidemiological research in Nigeria reported lower estimates of dietary sodium intake. For example, Odili et al. reported a median (interquartile range) urinary sodium excretion of 99 (105) mmol among 2,509 adults in 12 Nigerian communities.^6^ However, variability was wide in this study, and detailed dietary data were not collected to corroborate these urinary findings. Further, the 2023 World Health Organization report on dietary sodium intake estimated that daily sodium intake in Nigeria was 2,524 mg per day (95% uncertainty interval: 2,376, 2,673) based on modeling data.^1^ However, dietary sodium intake has been consistently underestimated in low- and middle-income countries in large modeling studies when compared with population-based sampling studies like this one.^23^ On the other hand, the 2019 baseline reported in the National Multisectoral Action Plan for the Prevention and Control of Noncommunicable Diseases was higher at 10 g of salt per day^5^, compared with approximately 9.5 g of salt per day in the current study. Thus, the current results likely provide a closer estimate of the true dietary sodium intake level in Nigeria than previous studies.

These results have important policy implications as Nigeria seeks to implement the World Health Organization SHAKE technical package through the National Multisectoral Action Plan for the Prevention and Control of Noncommunicable Diseases.^5^ Policy priorities outlined in the Action Plan include mandatory sodium limits in packaged foods, front-of-package labeling, and mass media and education campaigns to raise awareness about healthy diets, which align with the SHAKE package. These policies are cost effective^24^ and are considered appropriate by many stakeholders, especially given the high sodium levels and incomplete sodium labeling in packaged foods in Nigeria.^12^ On the other hand, barriers to SHAKE package policy implementation include low awareness of the harms of excess sodium intake, high cost of healthier foods, and taste preference for higher sodium foods.^13^ In contexts where discretionary salt use is high, like Nigeria, additional policy strategies may be considered to better match the sources of sodium through multisectoral stakeholder engagement.^25^ For example, potassium-enriched salts are effective, cost saving strategies to reduce blood pressure, total mortality, cardiovascular disease mortality, and cardiovascular disease events.^26,27^ Potassium-enriched salts could be promoted or subsidized, including in high-sodium products such as bouillon, as policy options to complement other SHAKE package strategies. Health education and mass media strategies may further to help to increase knowledge and awareness about this and other evidence-based interventions to reduce excess dietary sodium intake.

This study included numerous strengths, including a large, population-based sampling frame in a setting where dietary sources of sodium have not been rigorously assessed. Additional strengths include training and certification of dietitians for high quality data collection, adaptation of the NDSR for the Nigerian context, multiple pass dietary data collection with concurrent 24-hour urine collection, evaluation of location of food source, and a high proportion of repeated dietary recalls collected.

This study also has some limitations. First, participation among females was higher than among males, which may be due to the household recruitment approach. Therefore, given the observed sex differences in dietary sodium intake, the study sample likely underestimates the true dietary sodium intake on the population level. Second, data were collected in only three of 36 states in Nigeria, which may limit the generalizability of the findings. However, these states are geographically distributed through Nigeria, and we observed significant differences between states that may be relevant to those respective regions. However, to mitigate both limitations, we conducted analyses adjusted for the Nigerian population. Third, data were collected in a two-month period. We collected data on both weekdays and weekends to account for short-term temporal variability based on other studies but may have missed longer-term seasonal trends. On the other hand, this study is the first of three waves of population surveys to estimate dietary sodium intake in Nigeria, and future results may refine these results. Fourth, results from 24-hour urine collection were generally lower than those from dietary recalls. This may be due, at least in part, to under-collection of urine samples. However, we conducted sensitivity analyses using numerous published thresholds for urine volume and creatinine and adjusted for insensible losses as recommended.^15^ Notably, in the Federal Capital Territory, where we standardized sodium excretion levels to 24 hours, the 24-hour urine sodium excretion closely aligned with dietary intake in males and exceeded dietary intake in females. Despite the lack of standardization in Kano and Ogun, we still show high levels of agreement using Bland Altman and difference-in-difference plots based on reporting recommendations.^17^ These findings may also be due to differences in sodium and potassium excretion among individuals of African descent compared with other populations.^28^

### Conclusions

Adults in the Federal Capital Territory, Kano State, and Ogun State consume nearly two times the recommended level of dietary sodium recommended by the World Health Organization, largely from home cooked foods, nearly half of which comes from discretionary sources. This excessive intake contributes to the large and growing burden of hypertension and hypertension-related diseases in Nigeria, underscoring the need for targeted interventions. These findings have important policy implications for the implementation of the SHAKE package, including the potential benefits of promoting the use of potassium-enriched salts.

## Supplementary Material

Supplement 1 Tables 1 to 3 are available in the Supplementary Files section

## CLINICAL PERSPECTIVE

- This is first dietary sources of sodium study in Nigeria, which uses robust methods to inform dietary sodium policymaking.
- Results show an adjusted median sodium intake of 3,803 mg per day among Nigerian adults, which is nearly two times higher than recommended by the World Health Organization and much higher than has been previously reported.
- Nearly half (48.7%) of dietary sodium consumed in Nigeria comes from discretionary sources, including 21.4% from bouillon alone, both of which are much higher than in the United States.
- Nigerian adults with or at risk for hypertension may benefit from strategies that reduce discretionary sodium intake, such as potassium-enriched, low sodium salts or bouillon.
- Nigerian sodium policymaking can incorporate these results to achieve national targets related to reducing excess dietary sodium intake.
