# Supplementary material for: Dietary Sources of Sodium in Nigerian Adults From 3 Geographic Regions: A Population-Based Cross-Sectional Study
Source: Res Sq. 2025 Jan 15:rs.3.rs-5829587. Preprint. [Version 1] doi: 10.21203/rs.3.rs-5829587/v1 (PMC11774465; doi:10.21203/rs.3.rs-5829587/v1)
Supplement: Supplement 1 — Tables 1 to 3 are available in the Supplementary Files section [file NIHPPRS5829587v1-supplement-1.pdf]

Supplementary Files

This is a list of supplementary files associated with this preprint. Click to download.

- Tables.docx
- NaSSSourcesofSodiumReportWave1Supplemental20240701forCirculation02.08.24.docx
